# An open chat with…Beáta Vertessy

**DOI:** 10.1002/2211-5463.13077

**Published:** 2021-02-11

**Authors:** Ioannis Tsagakis, Beáta Vertessy, Duncan Wright

**Affiliations:** ^1^ FEBS Open Bio Editorial Office Cambridge UK; ^2^ Institute of Enzymology Research Centre for Natural Sciences Budapest Hungary

## Abstract

As *FEBS Open Bio* approaches its 10th anniversary, we have decided it is an ideal time to give the members of our prestigious Editorial Board an opportunity to discuss their research, publishing, and the activities of FEBS in the virtual pages of the journal. This new interview series is being helmed by Professor Beata Vertessy, who has been on the *FEBS Open Bio* Editorial Board since 2013 and has recently been promoted to Senior Editor. As the organizer, she has agreed to give the inaugural interview.

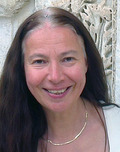

Beáta G. Vértessy was born in Budapest, Hungary. She obtained M.Sc. degrees in chemical engineering from the BME Budapest University of Technology, Hungary, in 1984, and in molecular biology from the University of Chicago in 1987, Ph.D./C.Sc. from the Eötvös Loránd University and the Hungarian Academy of Sciences in 1991, and D.Sc. from the Hungarian Academy of Sciences in 2001. Her laboratory (http://biostruct.org) focusing on Genome Metabolism and Repair at the Institute of Enzymology, Budapest, Hungary, was started in 2000 based on funding from the Howard Hughes Medical Institute, U.S.A., Wellcome Trust, U.K., and Alexander von Humboldt‐Stiftung, Germany. In 2010, she also started a structural biology laboratory at the BME Budapest University of Technology and Economics. The current research in her group aims to understand prevention, recognition, and repair of uracil in DNA from perspectives of structural and cell biology.

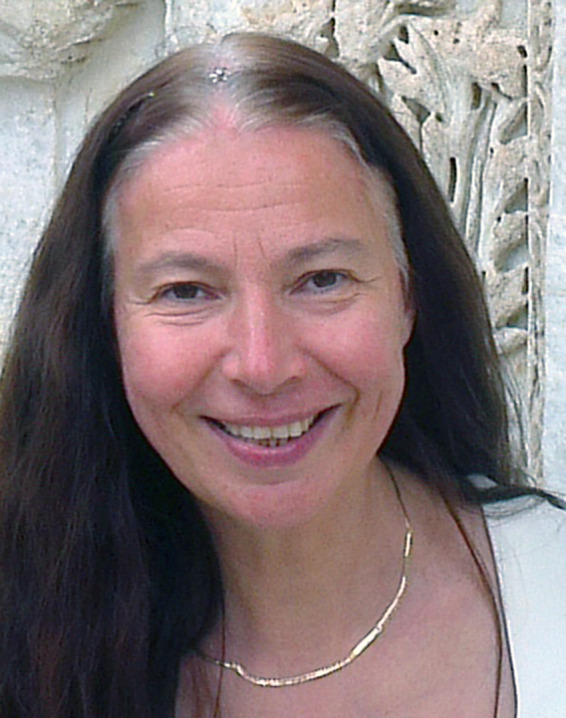

## For those who are nonspecialists, how would you describe your research?

Our research aims to understand the as of yet undiscovered details of genomic chemistry. The textbook concept that DNA consists of four bases is clearly not valid: several rarer but highly important bases are also present in DNA, and these play key roles in the regulation of normal and cancer cells. Our research is expected to reveal novel pathways that will allow us to specifically target cancer cells while causing minimal harm to normal cells.

Our research is also relevant in the context of infectious diseases such as tuberculosis and malaria, since DNA‐based regulatory pathways in pathogenic microbes are of great significance for biomedicine.

## How did your fascination with the metabolism of uracil‐substituted DNA start and where has that led your research?

Throughout my undergraduate studies and my MSc in chemical engineering, the chemical focus was of course very prominent. Quantitative aspects and clear logical thinking were also at the centre of my studies, and such training was highly beneficial for my later research in biochemistry and molecular biology. Uracil and thymine nucleobases only differ by a single methyl group, and it is fascinating to understand the different occurrences of these two bases in RNA versus DNA. It is also quite extraordinary that this ‘simple’ methyl substitution between uracil and thymine requires an arsenal of metabolic pathways due to the inherent chemical challenge of introducing a methyl group onto the pyrimidine ring. Because of this chemical challenge, there is a multifaceted competition for activated methyl groups in cellular metabolic pathways from nucleotide biosynthesis to amino acid biosynthesis and epigenetic pathways.

When I started working in the uracil–DNA field, it was already known that DNA could contain uracil either via thymine replacing misincorporation or via deamination of cytosine. However, it was highly disputed whether or not uracil in DNA had any physiological role. We first showed the significance of uracil‐DNA in the development of *Drosophila* (fruitfly) and then aimed to characterize the quantitative patterns of uracil genomic distribution in different models, a task which required us to develop new tools. Our recent identification of uracil–DNA patterns revealed an intriguing new mechanism for drug action in human cancer cells [1]

## What are some limitations in your field of research? How are people trying to overcome these?

A true and valid understanding of DNA chemistry is paradoxically and unfortunately greatly impeded by next‐generation genome‐wide sequencing technologies. This may sound counterintuitive, but the reason for this is that the vast majority of DNA sequencing methods rely on reading the sequence of bases as they are incorporated into the newly synthesized DNA strand following Watson‐Crick base pairing rules. In these methods, only the four canonical DNA building blocks (dATP, dGTP, dTTP, and dCTP) are used, so the readout will also be limited to these four nucleotides with adenine, guanine, thymine, and cytosine bases.

However, let’s consider a situation where the template strand contains both cytosine and also a noncanonical analogue, for example, methyl‐cytosine. Both cytosine and its methyl‐derivative will pair with guanine, so the sequencing readout will hide this difference. Similarly, both thymine and uracil base pair with adenine—again, sequencing is unable to make this distinction, and therefore, the readout fails to reveal key information on template strand composition and sequence. Clever chemical solutions and biotechnology advances are required to decipher the true DNA sequence, and numerous research groups, including ours, work on these problems.

## You moved around quite a bit during your training and early career—what stimulated your choices of institution during that time and how did these choices help shape your future research focus?

For a successful scientific academic career, it is indispensable to learn and acquire experience in various research laboratories and on multiple projects to develop the key competencies required. For me, it was of great importance to undertake highly diverse projects from metabolic regulation and erythrocyte membrane elasticity to nucleotide metabolism and DNA damage and repair. A life‐changing grant from the Howard Hughes Medical Institutes then allowed me to start the uracil in DNA project in my own laboratory. To tackle research questions on uracil–DNA, my laboratory uses diverse methodologies from structural biophysics to molecular and cell biology—all of which are techniques I previously gained experience in during my earlier postdoctoral positions.

## You also chair the FEBS Advanced Courses Committee and are an *ex officio* member of the FEBS Network and FEBS Education Committees. How do your roles on these committees help you support early career researchers?

FEBS aims to facilitate the careers of young scientists through multiple avenues. For the Advanced Courses, where the fields cover hot topics in molecular life sciences, our major goal is to allow the young scientist to make personal contacts with the leaders of any given field. We also look for feedback from the participants so that we can further improve these events.

The Network is a great platform on which to learn and socialize—especially in the current pandemic situation. The Education events also focus on career planning and aim to address many key areas required to build a successful career.

## What’s your proudest achievement outside of science?

Family—together with my husband, we have two children and five grandchildren, and it is so much joy to see how they grow and develop. It is also a great happiness for me to work together with many young people in my laboratory and combine education and research. We are really a family‐friendly environment in my group—to date more than 20 babies have been born to my students while they were working in my laboratory.
